# Preclinical activity of melflufen (J1) in ovarian cancer

**DOI:** 10.18632/oncotarget.11163

**Published:** 2016-08-09

**Authors:** Charlotte Carlier, Sara Strese, Kristina Viktorsson, Ebba Velander, Peter Nygren, Maria Uustalu, Therese Juntti, Rolf Lewensohn, Rolf Larsson, Jack Spira, Elly De Vlieghere, Wim P. Ceelen, Joachim Gullbo

**Affiliations:** ^1^ Department of Surgery, Laboratory of Experimental Surgery, Ghent University, Ghent, Belgium; ^2^ Department of Medical Sciences, Division of Cancer Pharmacology and Computational Medicine, Uppsala University, Uppsala, Sweden; ^3^ Department of Oncology and Pathology, Karolinska Biomics Center, Karolinska Institutet, Stockholm, Sweden; ^4^ Department of Immunology, Genetics and Pathology, Uppsala University, Uppsala, Sweden; ^5^ Oncopeptides AB, Stockholm, Sweden; ^6^ Present address: InSpira Medical AB, Tyresö, Sweden; ^7^ Radiation Oncology and Experimental Cancer Research, Laboratory of Experimental Cancer Research, Ghent University, Ghent, Belgium

**Keywords:** ovarian cancer, melflufen, preclinical, in vivo, intraperitoneal treatment

## Abstract

Ovarian cancer carries a significant mortality. Since symptoms tend to be minimal, the disease is often diagnosed when peritoneal metastases are already present. The standard of care in advanced ovarian cancer consists of platinum-based chemotherapy combined with cytoreductive surgery. Unfortunately, even after optimal cytoreduction and adjuvant chemotherapy, most patients with stage III disease will develop a recurrence. Intraperitoneal administration of chemotherapy is an alternative treatment for patients with localized disease. The pharmacological and physiochemical properties of melflufen, a peptidase potentiated alkylator, raised the hypothesis that this drug could be useful in ovarian cancer and particularily against peritoneal carcinomatosis. In this study the preclinical effects of melflufen were investigated in different ovarian cancer models. Melflufen was active against ovarian cancer cell lines, primary cultures of patient-derived ovarian cancer cells, and inhibited the growth of subcutaneous A2780 ovarian cancer xenografts alone and when combined with gemcitabine or liposomal doxorubicin when administered intravenously. In addition, an intra- and subperitoneal xenograft model showed activity of intraperitoneal administered melflufen for peritoneal carcinomatosis, with minimal side effects and modest systemic exposure. In conclusion, results from this study support further investigations of melflufen for the treatment of peritoneal carcinomatosis from ovarian cancer, both for intravenous and intraperitoneal administration.

## INTRODUCTION

Ovarian cancer (OC) has the highest mortality rate of all gynecological malignancies in western countries [1, 2]. In contrast to other common solid cancers, it is mostly diagnosed at an advanced stage, due to a lack of clinical symptoms and effective screening methods [3]. Currently, optimal debulking surgery combined with intravenous (IV) carboplatin/cisplatin and paclitaxel-based chemotherapy is the standard of care for primary OC, but historically melphalan has been an important therapeutic option [4, 5]. Approximately 70–80% of patients with OC will relapse after first-line chemotherapy, and the majority of them will eventually die of their chemotherapy-resistant disease [6]. Therefore, new and more efficacious drugs for treatment of ovarian canacer are needed. However, as this disease rarely spreads systemically and remains mostly confined to the peritoneal cavity, and given that intravenous (IV) chemotherapy usually does not effectively penetrate into peritoneal tumors, locoregional administration has been investigated [7, 8]. Administration of intraperitoneal chemotherapy (IPEC), whether or not under hyperthermic conditions ((H)IPEC), might offer a benefit for peritoneal metastases as compared to IV chemotherapy, since it allows to achieve higher drug concentrations in the peritoneal fluid but less systemic toxicity [5, 8, 9].

Melflufen (melphalan flufenamide, chemically described as melphalanyl-p-L–fluorophenylalanine ethyl ester) is an optimized lipophilic and targeted derivative of melphalan, an alkylating agent used for the treatment of different cancers for over fifty years. The hydrolytic cleavage of melflufen into melphalan in the cancer cell is mediated by the action of aminopeptidases, like aminopeptidase N (APN) [10–12]. APN or CD13, is a transmembrane ectopeptidase which is overexpressed in several hematopoietic and solid malignancies including ovary-, breast-, lung-, and thyroid cancers [13]. Additionally, APN has been described as marker of a malignant phenotype and to regulate tumor cell invasion, differentiation, proliferation, and apoptosis, as well as angiogenesis [14–16]. Interestingly, tumor samples from OC patients showed ubiquitous expression of APN in tumor associated blood vessels that were common in serous or mucinous subtypes, but less often in clear cell epithelial subtype [17, 18]. Moreover, inhibition of APN was reported to suppress proliferative- and migratory abilities of OC cells [19]. A study by Surowiak *et al*. suggested that APN is expressed to a higher extent in tumor samples taken during primary surgery as compared to samples from interval debulking after neoadjuvant chemotherapy, indicating a decrease in APN expression after initial chemotherapy treatment [18].

Preclinical *in vitro* and *in vivo* models showed that melflufen exhibits significant antitumor activity in various cancers, superior to melphalan, as well as anti-angiogenic activity [13, 20–23]. Furthermore, melphalan has also been reported to be an efficacious alternative agent for patients undergoing cytoreductive surgery (CRS) and (H)IPEC, for aggressive and recurrent peritoneal surface malignancies [24, 25]. Since it is hypothesized that the lipophilicity of melflufen is balanced by its susceptibility to enzymatic hydrolysis, it is hypothesized that these properties make melflufen an ideal intraperitoneal (IP) agent for chemotherapy in OC patients. With this study, we want to investigate the effects of systemic as well as locoregional treatment with melflufen in preclinical models of OC.

## RESULTS

### Cytotoxic activity in OC cell lines and primary human tumor cells

**Figure 1:**
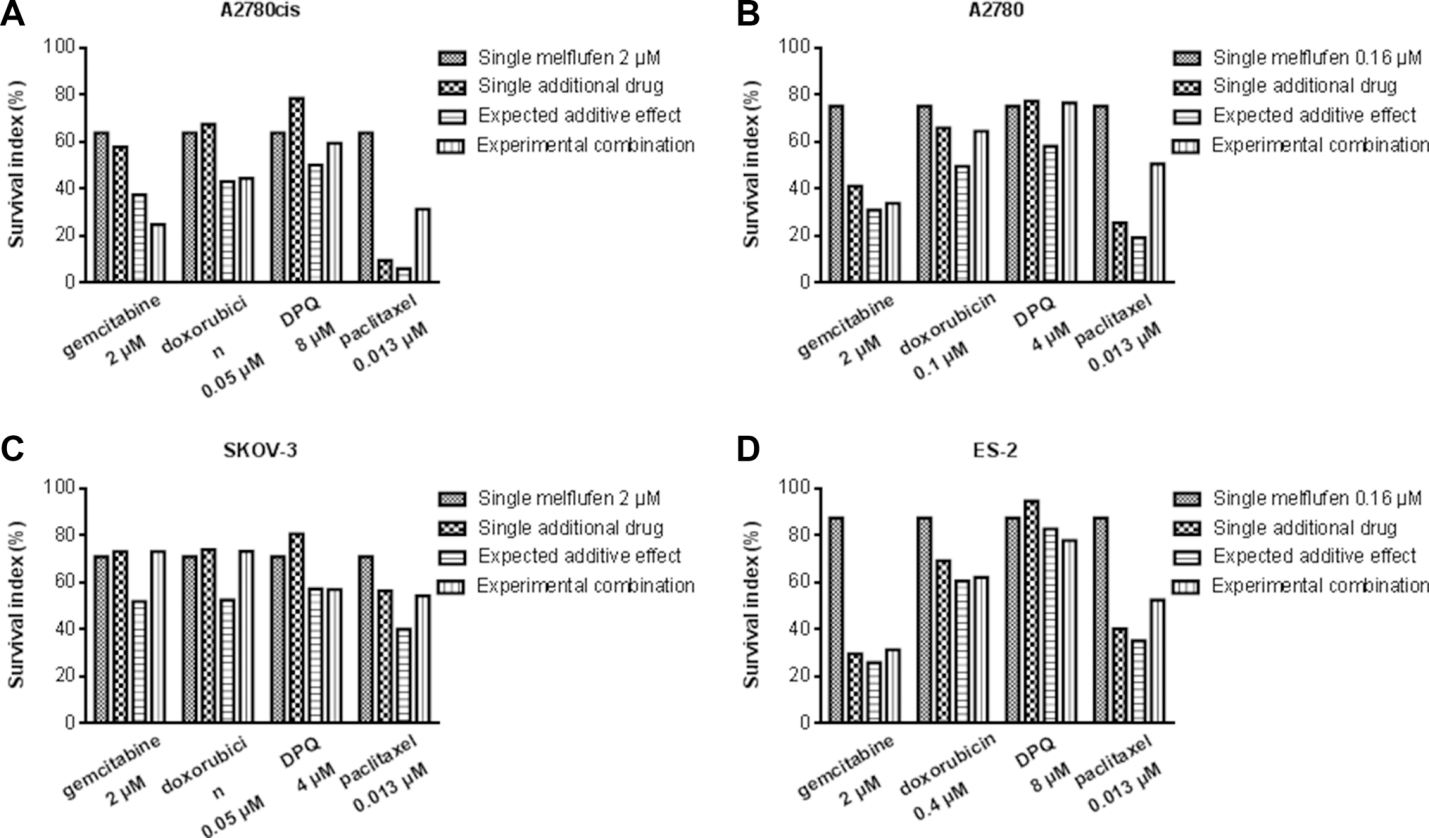
Melflufen combinations in different cell lines Survival index of melflufen (2 μM) alone or in combination with gemcitabine (2 μM), doxorubicin (0.05 μM), DPQ (8 μM) or paclitaxel (0.013 μM) in A2780, A2780cis, SKOV-3, and ES-2 cell line. Mean of 3 replicates.

Melflufen was active against OC cell lines A2780, A2780cis, ES-2, SK-OV-3 and SK-OV-3-Luc IP1 *in vitro*. The cytotoxic inhibitory concentration 50% (IC_50_) values were in the micromolar range (0.26–3.1 μM), and with a 5-32-fold superiority over melphalan (Table 1). Incubation of A2780 cells in hypoxic (1% O2) or anoxic (0.1% O2) environment reduced the activity of melflufen modestly, the IC_50_-value increased 1.2- and 2.0-fold respectively (not shown). For melphalan this effect was slightly more pronounced (2.0 and 2.6-fold, respectively) [26]. Melflufen in combination with carboplatin, doxorubicin, etoposide, gemcitabine, paclitaxel or the Poly-ADP (Adenosine Diphosphate)-Ribose Polymerase (PARP) III inhibitor DPQ, showed mainly additive effects, but also many antagonistic effects and some examples of synergistic events e.g. melflufen in combination with gemcitabine in a cisplatin resistant ovarian carcinoma cell line (A2780cis), and in combination with DPQ in ES-2. Doxorubicin combinations were usually additive. All tested combinations of melflufen and paclitaxel were antagonistic. A selection of melflufen combinations with gemcitabine, doxorubicin, DPQ and paclitaxel in A2780cis, A2780, SKOV-3 and ES-2 are shown in Figure 1.

**Table 1:** IC_50_-values and ratio of melphalan and melflufen in A2780, A2780cis, ES-2, SK-OV-3 (obtained with FMCA), and SK-OV-3 Luc IP1 (obtained with MTT) with 95% confidence intervals in parenthesis

Cell line	IC_50_(μM) melflufen	IC_50_ (μM) melphalan	IC_50_(μM) cisplatin	melphalan/melflufen
A2780	0.26 (0.14–0.48)	1.4 (0.45–4.4)	1.1 (0.38–3.3)	5
A2780cis	1.3 (0.93–1.8)	19 (7.7–46)	7.6 (2.5–23)	15
ES-2	0.29 (0.21–0.41)	2.0 (0.90–4.5)	2.1 (1.4–3.3)	7.0
SK-OV-3	3.1 (2.1–4.7)	> 100	52 (36–75)	> 32
SK-OV-3-Luc IP1	0.89 (0.47–1.7)	ND	12 (7.6–17)	ND

ND = Not determined.

**Figure 2:**
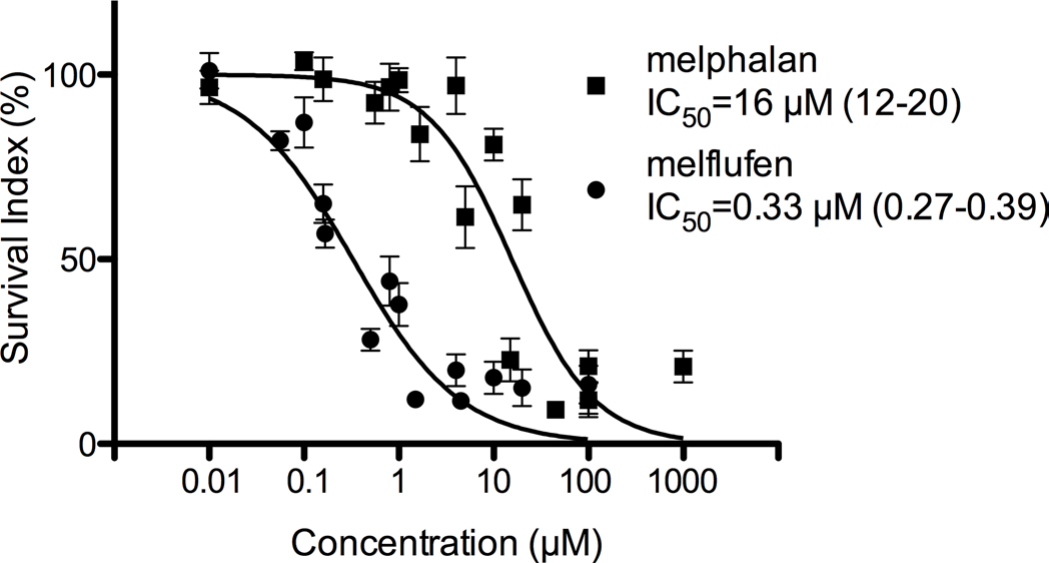
Activity of melflufen and melphalan in patient-derived OC cells The *in vitro* activity of melflufen (IC_50_= 0.33 μM) and melphalan (IC_50_ = 16 μM) in primary cultures of patient-derived OC cells (*n* = 82). Data are represented as mean ± SEM of 82 patient samples.

**Figure 3:**
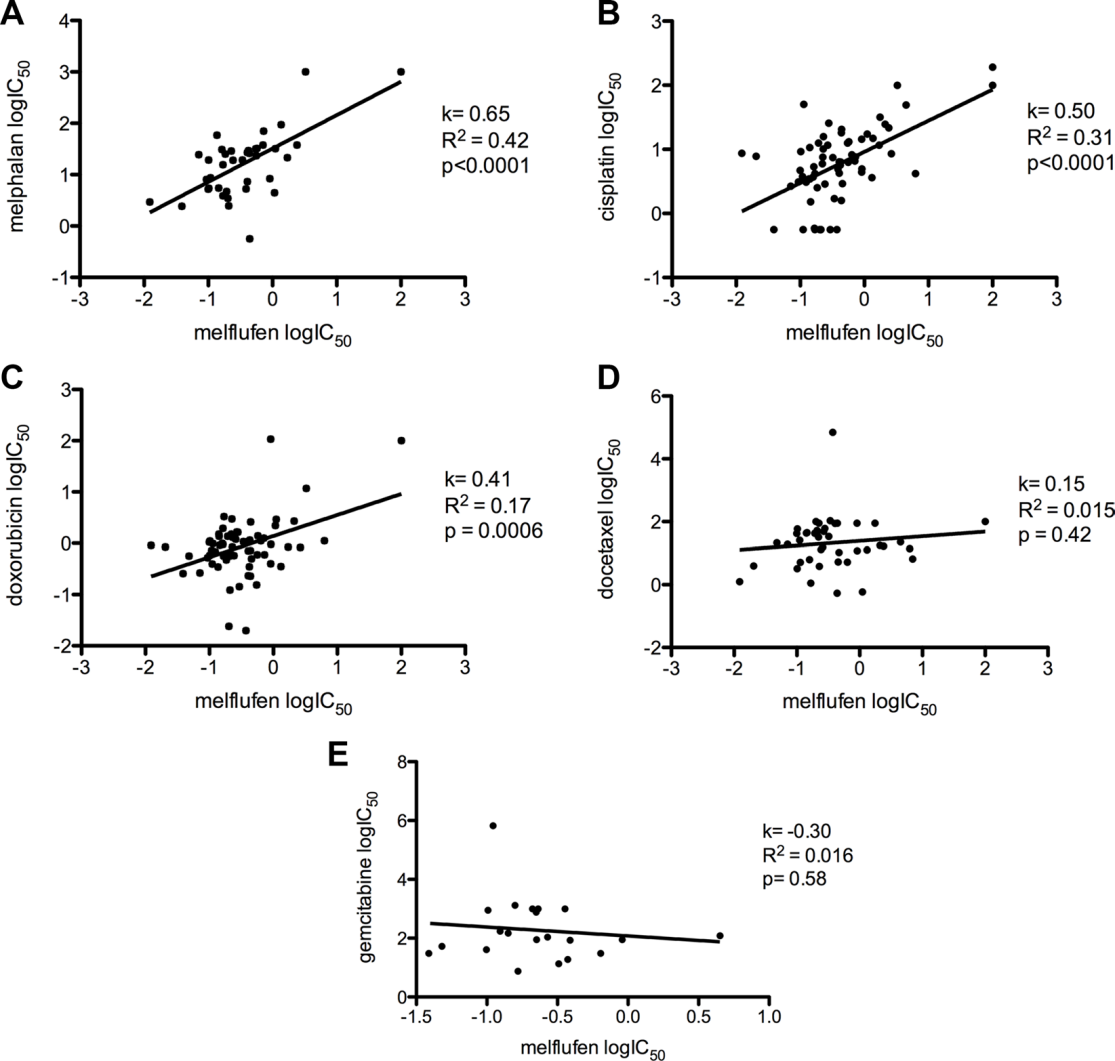
The correlation of melflufen and other tested drugs in patient-derived OC cells Melflufen vs. (**A**) melphalan (*R*^2^ = 0.42), (**B**) cisplatin (*R*^2^ = 0.31), (**C**) doxorubicin (*R*^2^ = 0.17), (**D**) docetaxel (*R*^2^ = 0.015) and (**E**) gemcitabine (*R*^2^ = 0.016), in primary cultures of patient-derived OC cells.

In primary cultures of patient-derived OC cells, the mean IC_50_–value for melflufen was 0.33 μM (95% CI of 0.27-0.39 μM) and for melphalan 16 μM (95% CI of 12–20 μM), i.e. a 49-fold potency difference (Figure 2). No significant results were obtained for the analysis of stratification factors in the patient material (ascitic effusion, histological classification, prior chemotherapy, response to subsequent chemotherapy, patient survival after sampling, or stage of disease at sampling). Interestingly, samples obtained from patients treated with Pt containing chemotherapy in their most recent therapy responded as well to melflufen as samples from previously untreated patients (IC_50_–value post platinum = 0.24 and *de novo* = 0.27 μM). The cytotoxic activity (expressed as logIC_50_) of melflufen vs melphalan was significantly positively correlated (*R*^2^ = 0.42, *p* < 0.0001), as expected for two alkylating agents. Furthermore, the activity correlated fairly well for melflufen and other DNA-interacting agents, i.e. vs cisplatin (*R*^2^ = 0.31, *p* < 0.0001), poorly but significantly vs doxorubicin (*R*^2^ = 0.17, *p* = 0.0006), but not vs docetaxel (*R*^2^ = 0.015, *p* = 0.42) or gemcitabine (*R*^2^ = 0.016, *p* = 0.58) (Figure 3).

### Melflufen IV shows *in vivo* activity in ovarian xenograft alone and combined with gemcitabine or liposomal doxorubicin

**Figure 4:**
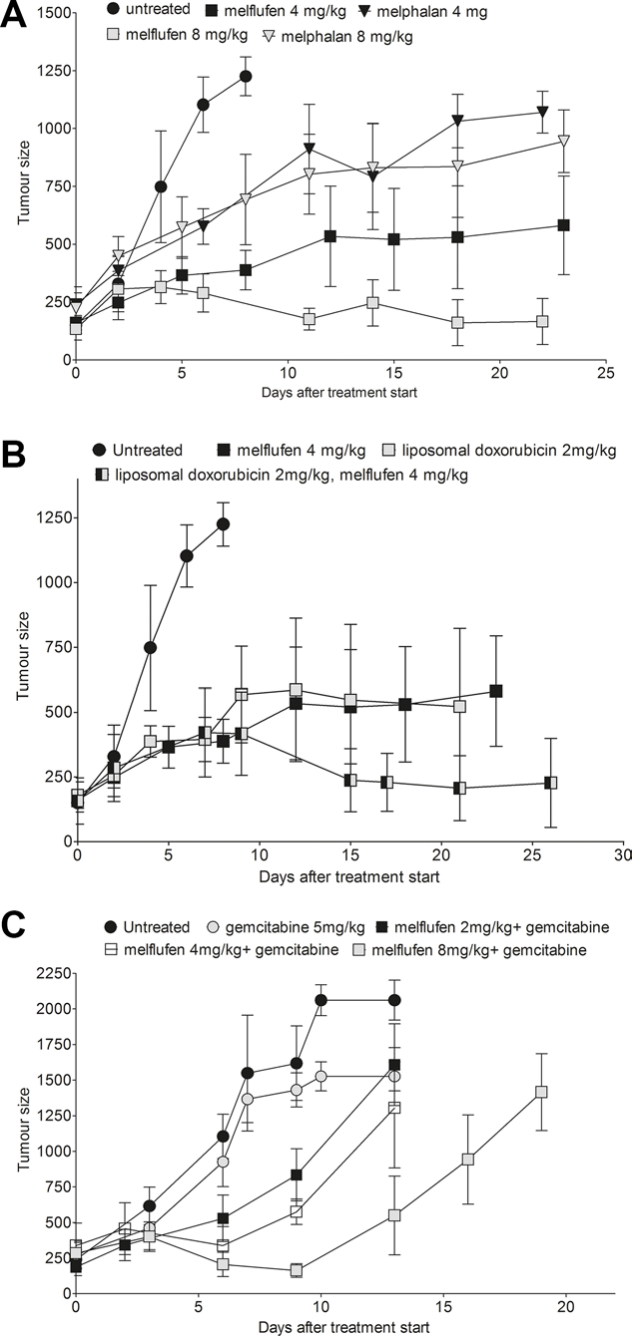
Intravenous melflufen in ovarian carcinoma xenograft alone or in combination with liposomal doxorubicin or gemcitabine Intravenous melflufen inhibits growth of ovarian carcinoma xenograft alone or in combination with liposomal doxorubicin or gemcitabine. Data shown are all mice (*n* = 4–5) at each time point, regardless if sacrificed or not. (**A**) Mean ± SEM of tumor size at indicated times post treatment with melflufen or melphalan (4 or 8 mg/kg). (**B**) Mean ± SEM of tumor size at indicated times post treatment with melflufen (4 mg/kg), also shown in (A) or liposomal doxorubicin (2 mg/kg) or their combination. (**C**) Mean ± SEM of tumor size at indicated time post treatment with gemcitabine (5 mg/kg) or gemcitabine (5 mg/kg) + melflufen (2, 4 or 8 mg/kg).

Comparing A2780 subcutaneous (SC) xenograft growth in untreated mice with those treated with single agent melflufen or melphalan (Figure 4A) showed that melflufen 8 mg/kg was significantly superior to the no treatment as well as to melphalan treatment with 4 mg/kg and 8 mg/kg (*p* = 0.0026). The effect of the combination of melflufen (4 mg/kg) with liposomal doxorubicin (2 mg/kg) was evaluated in the same A2780 ovarian carcinoma xenografts (Figure 4B). The combination offered better tumor control than either drug alone with almost no net tumor growth over the treatment period. The anti-tumor effect was significant (*p* = 0.0204), and multiparametric testing showed a significant difference between no treatment and the melflufen- liposomal doxorubicin combination. The effect of adding a sub-active dose of gemcitabine (i.e. 5 mg/kg, determined in a preparatory experiment) to melflufen 2, 4 or 8 mg/kg was also evaluated in A2780 xenografts (Figure 4C). Increased inhibition of growth of A2780 ovarian xenografts was evident after all melflufen doses with the highest dose approaching statistical significance (*p* = 0.0766). In a pilot experiment, the *in vivo* effect of melflufen was also evaluated in cisplatinum resistant A2780cis cells subcutaneously xenografted to female SCID mice. As expected, based on the observed *in vitro* cross resistance, neither melflufen (8 mg/kg) nor gemcitabine (5 mg/kg) had any significant effect on tumor growth. However, the combination of these two treatments resulted in delayed tumor growth and approximately doubled the time before reaching the endpoint, although with significant toxicity in the animals (not shown).

### *In vivo* activity of melflufen in IP and SP OC xenografts

**Figure 5:**
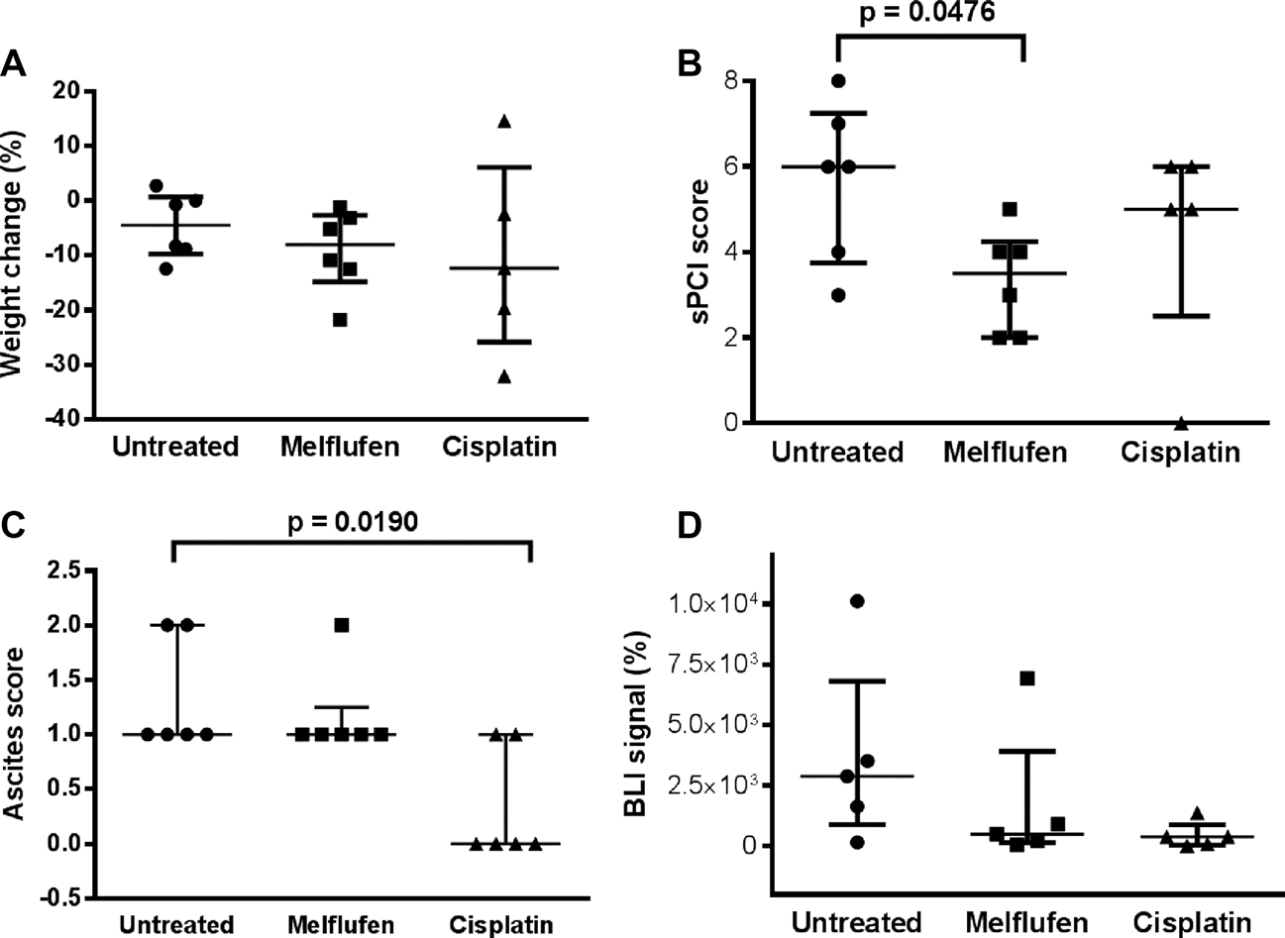
IP model: *in vivo* effects of melflufen and cisplatin in SK-OV-3 Luc IP1 xenografts Weight change during treatment (**A**); sPCI score **(B**); Ascites score (**C**); BLI signal, reported as relative value compared to the BLI signal at day 0 (**D**). (A, B and C) were measured when euthanized. Bars indicating median and interquartile range (*n* = 5 to 6 animals in each group).

In the IP xenograft model (Figure 5), the volume of ascites present at sacrification was significantly lower in the cisplatin-treated versus untreated mice (*p* = 0.0190). Moreover, the simplified peritoneal carciomatosis index (sPCI) score (measured as the number of regions affected by tumor deposits) was significantly lower in the melflufen-treated versus untreated mice (*p* = 0.0476). A non-significant trend towards a lower bioluminiscence imaging (BLI) signal was observed for both treated groups (melflufen and cisplatin, *p* = 0.688 and *p* = 0.143). No significant treatment related weight change was observed (*p* = 0.759 and *p* = 0.813, melflufen and cisplatin vs untreated mice, respectively), however, there was a non-significant trend for higher weight loss in the cisplatin treated animals. Also, 88% of the IP engrafted mice showed omental metastases.

**Figure 6:**
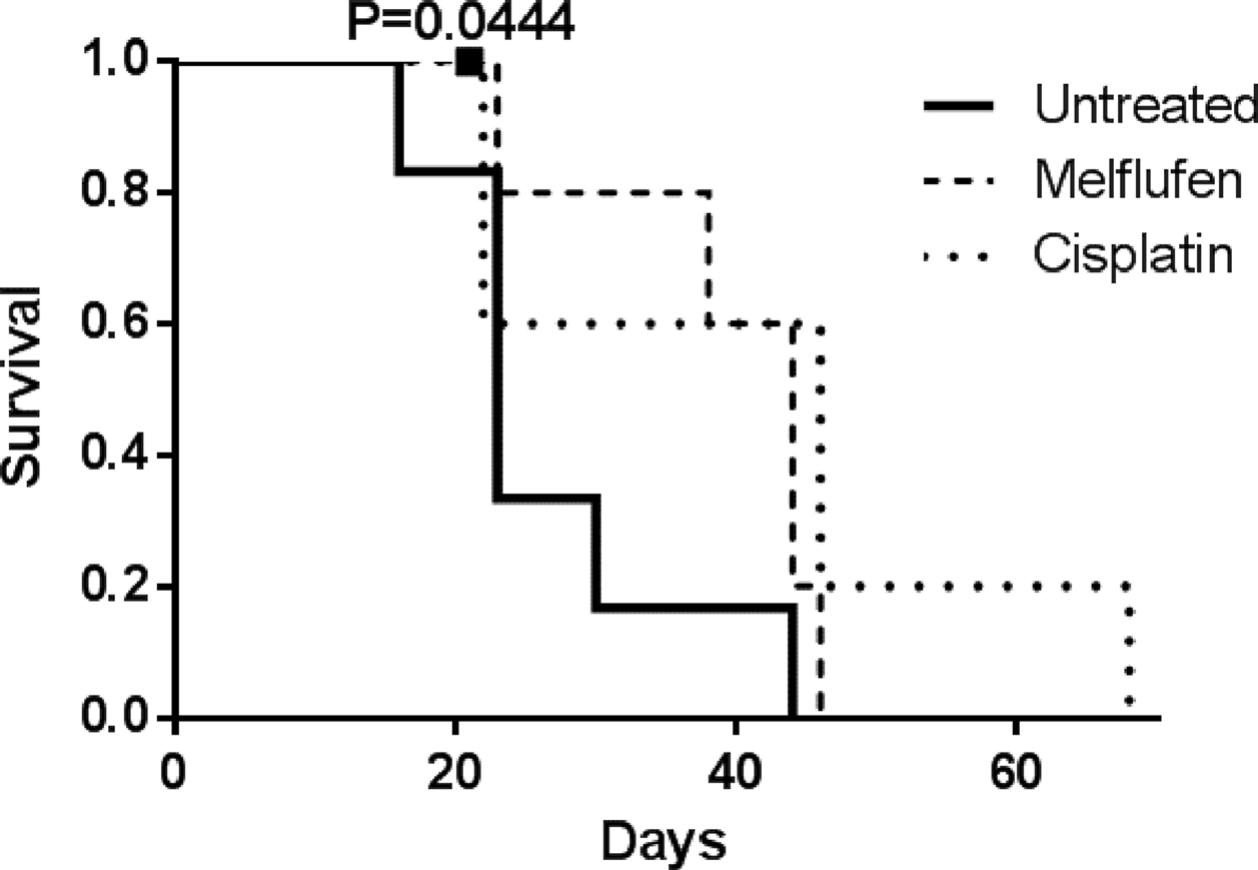
Kaplan-Meier survival curve of mice treated with melflufen and cisplatin Mice treated with cisplatin and melflufen live longer than untreated mice, after IP injection of SK-OV3-Luc IP1 cells. Two mice (overlap) were censored because of death from non tumor-related causes and were indicated with a square (*n* = 5 to 6 animals in each group).

Mice treated with melflufen and cisplatin showed a similar, median survival of 44 and 46 days, respectively, almost doubling the median survival of 23 days for untreated mice (Figure 6). By conducting the log-rank test for trend, the increased survival was found significant for the treated animals (*p* = 0.0444 for melflufen). Two mice were censored (one from the melflufen and one from the cisplatin group), due to the injection of the drug in the colon instead of IP, which leads to bacteria in the abdominal cavity and consequently to sepsis.

**Figure 7:**
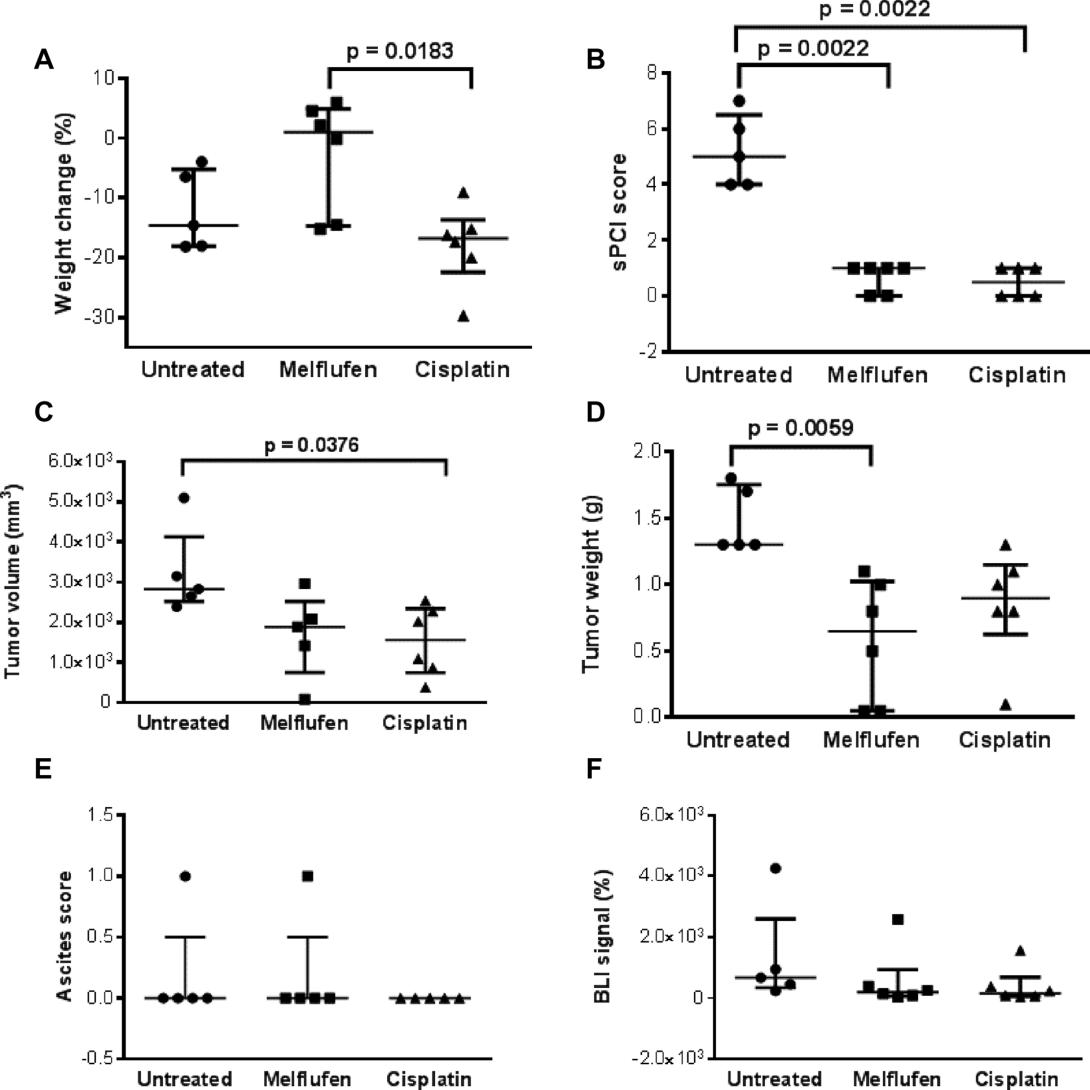
SP model: *in vivo* effects of melflufen and cisplatin in SK-OV-3 Luc IP1 xenografts Weight change during treatment (**A**); sPCI score (**B**); Tumor volume (**C**); Tumor weight (**D**); Ascites score (**E**); BLI signal, reported as relative value compared to the BLI signal of day 0 (**F**); (A–E) were measured when euthanized. Bars indicating median and interquartile range (*n* = 5 to 6 animals in each group).

In the subperitoneal (SP) xenograft model (Figure 7), weight loss of the cisplatin treated animals was significantly higher compared to the melflufen-treated group (*p* = 0.0183). Significant antitumor activity was observed in both treated groups, with a lower sPCI score as compared to the untreated group (both treated groups = 0.0022 vs control). Additionally, significant differences were demonstrated between the cisplatin-treated and untreated groups regarding tumor volume (*p* = 0.0376) and between the melflufen-treated and untreated group in terms of tumor weight (*p* = 0.0059). No significant differences were found regarding ascites score (*p* > 0.999) and BLI signal between the melflufen or cisplatin-treated and untreated mice, respectively (*p* = 0.372 and *p* = 0.175). Beside from the decreased weight loss there were no statistically significant differences between melflufen and the positive control cisplatin.

### Rapid uptake of melflufen in the IP cavity

Parmacokinetic analysis of melflufen and its metabolites in IP fluid or plasma in non-tumor bearing mice indicated that the peritoneal uptake of melflufen was extensive and rapid, as no remaining melflufen could be detected in IP fluid already at 5 minutes after administration (Table 2). Melflufen was added in a concentration of 73.3 μg/ml (corresponding to 136 μM) into the IP cavity. The metabolites, des-ethylmelflufen (de-esterification), and melphalan (peptide cleavage) were detected in the IP fluid at 5, 15 and 30 minutes at low concentrations (–8.8/9.7; 9.2/9.0; and 10/7.5 μM, respectively), corresponding to about 15% of the added dose. In plasma, only a small amount of melphalan could be detected (2.2 μM) at 30 minutes, and a very low concentration of des-ethylmelflufen (86 nM), which is consistent with the high esterase activity in rodent blood.

**Table 2:** Pharmacokinetic analysis of melflufen and its metabolites in IP fluid and in plasma, after IP injection

Time (minutes)	Melflufen (μM)	Des-ethyl-melflufen (μM)	Melphalan (μM)
Dose (*t* = 0)	137	-	-
5 IP fluid	-	8.4 (5.4–12)	5.5 (4.8–6.4)
15 IP fluid	-	8.8 (7.8–9.5)	5.1 (3.9–5.8)
30 IP fluid	-	9.5 (7.0–12)	5.8 (5.6–6.0)
30 plasma	-	0.15 (0.082–0.22)	1.5 (1.3–1.8)

The lowest and highest values are in parenthesis.

## DISCUSSION

Ovarian cancer (OC) carries a significant mortality, mainly related to a late disease stage at diagnosis [1–3]. Despite initial sensitivity to several lines of chemotherapy, in which platinum-taxane combinations play an important role, approximately two-thirds of patients eventually die from their disease. This illustrates the need for novel agents [6].

In this study, we demonstrate the *in vitro* cytotoxicity of melflufen against different OC cell lines and patient-derived OC cells, and show for the first time the *in vivo* antitumor efficacy of melflufen after IV or IP administration in different xenograft mouse models. The historical value of single agent melphalan in the treatment of OC is indisputable [4]. Melflufen, a lipophilic and targeted derivative of melphalan, is potentiated by the action of aminopeptidases such as APN, which is overexpressed or exhibits altered enzymatic activity in OC [11, 13, 27]. Melflufen showed a superior antitumor activity compared to melphalan in various *in vitro* and *in vivo* models of human cancer, including anti-angiogenic activity [13, 20–23]. The use of IP chemotherapy permits the administration of higher drug doses, while minimizing systemic toxicity [5, 8, 28–30]. Several large randomized trials have demonstrated that the addition of catheter-based IP chemotherapy to adjuvant IV treatment results in a significantly better survival compared to IV treatment alone [31–33]. The above considerations prompted us to investigate the preclinical effects of melflufen in different OC xenograft models, with cisplatin as a positive control treatment, since the platinum compounds are considered the standard drug for IP and IV treatment of OC [30, 34].

Results of our *in vitro* experiments confirm the superior antitumor activity of melflufen compared to melphalan, showing a 5 to 32-fold higher activity for OC cell lines and a 43-fold higher activity for primary cultures of patient-derived OC cells. In addition, the antitumor effect of melflufen was also demonstrated *in vivo* in different xenograft models with A2780 cells xenografted SC and SK-OV-3 Luc IP1 cells xenografted IP and SP. Primary human tumor cells (PHTC) have received relatively little attention as a preclinical cancer stem cell like model [35]. In fact, *in vitro* response analysis of PHTC reports accurate diagnosis specific activity of cancer drugs and predict clinical response for individual patients [36, 37]. Furthermore, the *in vivo* selection of cells able to establish tumors in the used SK-OV-3 Luc IP model selects cells with ovarian cancer stem cell-like properties.

Systemically administered melflufen showed single agent activity in a subcutaneous A2780 xenografts model. Furthermore, additional *in vitro and* xenograft studies revealed an improved anti-tumor activity and tumor control, respectively, when melflufen was combined with either gemcitabine or liposomal doxorubicin, two agents used commonly used in OC [38, 39]. Previously, our group found synergistic activity of melflufen and the DNA-topoisomerase II inhibitor etoposide against several cell lines [40]. In addition, IP administered melflufen showed also antitumor efficacy in intraperitoneal (IP) and subperitoneal (SP) SK-OV-3 Luc IP1 xenografts, based on the sPCI scores. The IP model was set up to mimic diffuse ovarian cancer, while the SP model was chosen to induce isolated tumor nodules that we could follow in time. In general, mice tolerated melflufen treatment very well, and only modest weight changes were observed, as previously reported by Gullbo *et al*. [22]. In contrast, cisplatin treated and untreated mice lost weight, probably due to dehydration and cancer cachexia, respectively [41, 42]. Remarkably, 88% of the IP xenograft mice showed omental metastases, which might be explained by the important role of omental milky spots and splenoportal fat [43]. Furthermore, a trend towards a lower BLI for both xenograft models was found. However, BLI data could not be easily interpreted due to distinct variability in relatively small groups and to camouflage of the total flux signal by the largest implant.

A median survival of 44 and 46 days was found for melflufen and cisplatin, respectively, compared to a median of 23 days in the control group. Finally, pharmacokinetic analysis after IP administration of melflufen indicated a rapid and complete uptake from the abdominal cavity with undetectable concentrations already after 5 minutes. Concentrations of 2.2 μM melphalan were detected in mouse plasma after 30 minutes, a time point suggested to be relatively close to the C_max_. Interestingly, this is considerably lower than the melphalan C_max_ of 50.3 μM after IP administration of the LD_10_ dose (71.3 mg/m²) [44]. This results suggest that the use of IP melflufen is safe and unlikely to yield mortality or significant hematologic toxicity. In contrast, systemically administered melflufen, given to patients with advanced or progressive ovarian carcinoma and non-small-cell lung cancer, caused reversible neutropeunia and thromocytopenia [27]. Paba-Prada *et al*. found the same dose-limiting toxicities in relapsed - refractory multiple myeloma patients treated with melflufen and dexamethasone [45].

In conclusion, we show a significant activity of melflufen *in vitro* against OC cell lines and primary cultures of patient-derived OC cells. Melflufen activity is superior compared to that of the parental drug melphalan in subcutaneous as well as intra- or subperitoneal OC xenografts.

In addition, synergism was achieved with gemcitabine and liposomal doxorubucin, possibly indicating a potential for combination therapy of these in clinical trials for patients where conventional therapies have failed.

## MATERIALS AND METHODS

### Ethics statement

Patient sampling for the primary cultures of human tumor cells (PHTC) was approved by the regional ethical committee (Dnr 237/2007).

All animal experiments were approved by the Animal Ethics Committee of the Faculty of Medicine at Ghent University (ECD 14/66) or by the Animal Ethical committee in Stockholm Sweden (N 284/08 to Dr Spira), and were performed according to Belgian, Swedish and European legislature on animal welfare. Mice were examined daily for pain or discomfort and “The Guidelines for the welfare and use of animals in cancer research” were strictly followed for distention of the abdomen, physical condition and other clinical signs of mice, which show necessary intervention, as prescribed by The National Cancer Research Institute (NCRI)” [46]. Food and water was given ad libitum. All procedures were performed under general anesthesia (Forene^®^, AbbVie, Waver, Belgium) and analgesia (Ketoprofen, 5 mg/kg).

### Reagents

The dipeptide melflufen, a kind gift from OncoPeptides AB, was dissolved in either dimethyl acetamide (DMA), dimetylsulfoxid (DMSO) or ethanol (EtOH) (Sigma-Aldrich, Diegem, Belgium or Stockholm, Sweden), stocked at –20°C and protected from light. Cisplatin Hospira was provided by Hospira Benelux BVBA (Antwerp, Belgium). Melphalan (Alkeran, GlaxoSmithKline, Mölndal, Sweden), gemcitabine (Gemzar, Lilly AB, Solna, Sweden), doxorubicin (Sigma-Aldrich) and liposomal doxorubicin (Caelyx, Schering-Plough, New Jersey, USA) were obtained from the State-owned Pharmacy Chain (Apoteket AB, Uppsala, Sweden), and the PARP III inhibitor, DPQ, was obtained from Merck Millipore (Solna, Sweden). All chemicals which were not obtained as infusion solutions were dissolved in EtOH or DMSO and diluted in phosphate buffered saline (PBS) or saline.

For the subcutaneous xenograft studies, melflufen or melphalan were dissolved in DMA and further diluted in 5% glucose infusion fluid into indicated final concentrations less than 15 minutes prior to injection. Liposomal doxorubicin and gemcitabine were also diluted in 5% glucose infusion fluid as described in Gallo *et al.* [47] to appropriate concentrations.

For the IP experiments, cisplatin and melflufen (dissolved in DMSO/EtOH) were diluted in saline less than 15 minutes prior to injection. Isofluran (Forene^®^), sevofluran (Sevorane^®^, AbbVie) and 1 mg/ml ketoprofen (Rofenid^®^, Sanofi Aventis, Diegem, Belgium) were used for anesthesia and analgesia of the animals. Glucose 5% (0.2 ml; B Braun Medical N.V., Diegem, Belgium) and Hartmann's solution (0.4 ml; B Braun Medical N.V.) were subcutaneously applied to hydrate all animals during treatment. Mice were IP injected with 0.2 ml (15 mg/ml) of D-luciferin (PerkinElmer, Zaventem, Belgium) 10 minutes prior to BLI, which was analyzed with the Living Image^®^ 4.3.1 software (IVIS^®^ Lumina II, PerkinElmer).

### Cell lines

The human OC cell lines, A2780 and A2780cis were obtained from Sigma Aldrich. The latter made resistant to cisplatin as previously described [48]. The ES-2 (CRL-1978) and SK-OV-3 (HTB-77) cells were both purchased from American Type Culture Collection (ATCC, Wesel, Germany). The cells were cultured in RPMI 1640 (A2780 and A2780cis) or McCoys 5A medium (ES-2 and SK-OV-3) and complemented with 10% FCS and 2% pest/glut (all Sigma Aldrich). The human OC cell line SK-OV-3-Luc IP1 is a more potent, luciferase positive OC cell line, created through *in vivo* selection [49]. Sort Tandem Repeat (SRT) profiling was conducted as described by De Vlieghere *et al*. [49]. This cell line was cultured in Dulbecco's Modified Eagle's Medium (DMEM, Life technologies, ThermoFisher, Ghent, Belgium), supplemented with 2% penicillin/streptomycin (Life technologies) + 0.005% fungizone (Bristol-Myers-Squib B.V., Utrecht, The Netherlands) and 10% FCS (Sigma-Aldrich) [50]. Saline and BD matrigel (Life Sciences, Antwerp, Belgium) were used to dilute SK-OV-3-Luc IP1 cells before IP and SP injection, respectively. The cell banks performed authentications by short tandem repeat analysis. All cell line experiments in Sweden were performed within 6 month after resuscitation, in Belgium STR was done to verify identity.

### Analyses of drug combinations

Additive combination effect analysis was performed at concentrations of melflufen and standard drugs, producing a viability of 60–80% in single drug analysis. Firstly, single drug activity was determined in the cell lines to select suitable combination concentrations. A fixed concentration of melflufen (0.32, 0.16, or 0.08 μM for A2780 and ES-2 and or 4, 2, or 1 μM for A2780cis and SK-OV-3) and a seven step dilutions concentration range for the standard drug was added to the cells and analyzed by the FMCA. The effect of the combination was defined as synergistic if the observed viability was lesser than the sum of both drugs administered alone, i.e. the expected additive effect, and antagonistic if it was greater than the sum of both drugs administered alone.

### Primary cultures of human tumor cells (PHTC) from patients

Ovarian cancer patient samples were obtained from 82 patients by either surgery or as needle biopsies, and delivered to the laboratory within 24 hours. The cell preparation protocol described by Blom *et al.* was used [51]. Briefly, the samples were minced and dispersed in collagenase (Collagenase type I, 1.5 mg/ml; Sigma and DNase Type I, 100 μg/ml; Sigma in CO_2_-Independent Medium; Gibco, pH 7.35 to 7.45) in 37°C for 1-4 hours and purified by density gradient centrifugation (histopaque®-1077; Sigma Aldrich). Viability was determined by staining the cells with trypan or toluidine blue and counting in a Bürker chamber. A cytopathologist estimated the proportion of tumor cells by inspection of May–Grunwald–Giemsa-stained cytospin preparations. The cells were diluted in RPMI 1640 (complemented with 10% FCS and 2% pest/glut) prior to seeding in culture plates for drug sensitivity testing.

For analysis of different groups, the patient material was stratified for the factors ascitic effusion (yes/no), histological classification (high grade serous/low grade serous/others), prior chemotherapy (yes/no), response to subsequent chemotherapy (complete response vs. partial response/stable disease vs. progressive disease), patient survival after sampling (none vs. less than 2 year) and stage of disease at sampling (I–IIIC vs IV).

### Viability assays (FMCA and MTT)

The Fluorometric cytotoxicity assay (FMCA) was used to assess the viability of OC cell lines and patient human tumor cells (PHTCs) as previous described elsewhere [52, 53]. Briefly, the drugs and the cells were seeded (5000 cells/well for both cell lines and PHTCs) into a 384-well microtiter plate (Nunc) using an Echo 550 liquid handler (Labcyte), and a pipetting robot Biomek 2000 (Beckman Coulter), respectively. The plates were incubated for 72 hours in a 37°C humidified atmosphere containing 5% CO_2_ and were then washed with PBS once, and 10 μg/ml FDA (50 μl) was added. After 60 minutes of incubation, fluorescence was measured (485/538 nm exitation/emission) in a Fluostar Omega fluorometer (Biotech). The fluorescence signal is directly proportional to the number of viable cells. Cell survival is presented as Survival Index% (SI%), defined as mean signal of each drug concentration divided by mean signal of the untreated cells (with blank signal subtracted). A concentration curve was made using Graph Pad Prism, and an IC_50_-value was calculated for each drug. Quality criteria for a successful assay included > 75% starting cell viability (by trypan or toluidine blue exclusion), a signal in untreated cells that were more than ten times the blank, and a coefficient of variation in untreated and blank wells of < 30%. Additionally, the effect of melflufen and melphalan was assessed in the A2780 cell line, cultivated in oxygen deprived environment (i.e. anoxia: 0.1% O_2_ or hypoxia: 1.0% O_2_) employing a Ruskinn InVivo_2_ 500 hypoxic incubator (Ruskinn Technology Ltd, Pencoed, UK) as previously described by Strese *et al*. [26].

In addition, prior to the IP *in vivo* experiments the traditional *in vitro* MTT ([3-4,5-Dimethylthiazolyl-2]-2,5-Diphenyltetrazolium Bromide; (Sigma-Aldrich)) cell viability assay was carried out, to determine and to compare the sensitivity (IC_50_) of the SK-OV-3 LUC IP1 cells to cisplatin. Briefly, SK-OV-3 Luc IP1 cells were seeded as monolayers in 96-well plates at a density of 4.0 × 10^4^ cells/ml. Subsequently, after 24 hours of incubation (37°C, 10% CO_2_), cells were exposed for 2 hours with cisplatin or melflufen at indicated concentrations (0.1-100 μM and 0.01-7 μM, respectively). After 72 hours of incubation, 20 μl MTT (5 mg/ml) was administered to each well for 2 hours. Subsequently, MTT solutions were removed and 100 μl of DMSO was added to each well to measure the absorbance of metabolically active cells. These measurements were performed at 570 nm in a Paradigm Detection platform and analysed with the Soft Max Pro 6.1 software (BIO-RAD laboratories, Hemel Hempstead, United Kingdom). Three independent MTT assays, with three replicates were performed.

### *In vivo* studies

### Subcutaneous xenografts

For the subcutaneous (SC) xenografts studies, A2780 cells were engrafted into immunocompromized mice. Female SCID mice with a BALB c background (MTC Animal Facility, Karolinska Institutet, Stockholm, Sweden) aged of 8-10 weeks were used. To establish the xenografts, 1.0×10^6^ A2780 cells were suspended in PBS and injected in a total volume of 0.2 ml subcutaneously in the right flank of each mouse. The animals were either left untreated or treated with melflufen, melphalan, liposomal doxorubicin or gemcitabine as indicated below. Tumor growth was measured by caliper at indicated time points. Tumor size was calculated by the formula: Tumor size = [length × width²]/ 2 as described by Gallo *et al*. [47]. Detailed treatment schedule of the subcutaneous xenografts is given in Table 3.

**Table 3:** Treatment schedule of subcutaneous xenografts

Designation	Route	Dose	Regimen
Vehicle	IV	-	2QW × 3W
Melflufen	IV	4 mg/kg	2QW × 3W
Melflufen	IV	8 mg/kg	2QW × 3W
Melphalan	IV	4 mg/kg	2QW × 3W
Melphalan	IV	8 mg/kg	2QW × 3W
Liposomal doxorubicin	IV	2 mg/kg	1QW × 3W
Liposomal doxorubicin + melflufen	IV + IV	2 mg/kg + 4 mg/kg	1QW × 3W + 2QW ×3W
Gemcitabine	IP	5 mg/kg	2QW × 2W
Gemcitabine + melflufen	IP + IV	5 mg/kg + 2 mg/kg	2QW × 2W
Gemcitabine + melflufen	IP + IV	5 mg/kg + 4 mg/kg	2QW × 2W
Gemcitabine + melflufen	IP + IV	5 mg/kg + 8 mg/kg	2QW × 2W

### Intraperitoneal and subperitoneal xenografts

Athymic, nude-foxn1nu female mice (ENVIGO, NM Horst, the Netherlands) of 6 weeks age and an avarage weight of 20 g were conditioned one week before the start of each study. Two OC xenograft mice models with SK-OV-3 Luc IP1 cells were employed to study the antitumor efficacy of IP administered melflufen compared with treatment using saline and cisplatin, respectively. Engraftment was made with tumor cells injected IP (2.0 × 10^6^ SK-OV-3 Luc IP1 cells, dissolved in 0.5 ml of saline) or bilateral in the SP space (between the muscle and the mesothelial layer of the peritoneal wall, 5.0 × 10^5^ of SK-OV-3 Luc IP1 cells dissolved in 50 μl BD matrigel (Life Sciences) [50, 54]. Mice (5-6/group) were treated IP, 1 or 3 weeks after the injection of SK-OV-3 Luc IP1 cells in the IP and SP model, with either melflufen or cisplatin and monitored for another two weeks after treatment (Table 4). Untreated animals were injected with only saline. After each treatment mice were imaged using BLI and this signal was reported as relative values compared to the BLI signal at day 0. Furthermore, the physical condition of mice was followed-up during and after treatment. After 2 weeks of follow-up, mice were sacrificed and tumor nodules were harvested, weighted and measured. Additionally, tumor volumes (mm^3^) were estimated according to the formula mentioned above [47]. The sPCI score was based on the number of affected regions, because individual tumor nodules were too small and too numerous. However, this method is clinically used and described elsewhere [55, 56]. In addition, the used sPCI score was in agreement with Van Der Vange *et al*. [57].

**Table 4:** Intraperitoneal treatment schedule of IP and SP xenografts

Xenograft model	Drug	Dose	Regimen
IP	Saline	-	3QW ×2W
IP	Melflufen	4 mg/kg	3QW ×2W
IP	Cisplatin	3 mg/kg	3QW ×2W
SP	Saline	-	3QW ×2W
SP	Melflufen	4 mg/kg	3QW ×2W
SP	Cisplatin	3 mg/kg	3QW ×2W

Animals were euthanized when a body weight loss of 20% at any timepoint or 15% maintained for 72 h was observed compared with the pre-treatment weight or with the age-matched controls. In addition, a human endpoint was set on a BLI signal of 1.0 × 10^11^ photons/second, considered to indicate maximal tumor growth. When sacrificed, autopsies were done for all mice and the number of affected sPCI areas were determined [57].

In addition, a survival experiment was set-up separately from the IP and SP tumor xenografts experiments. Therefore, the same IP model was used including treatment, imaging, and endpoints.

### Pharmacokinetic study

A pharmacokinetic study was performed in 6 non-tumor bearing animals treated once a week for 2 weeks (1QW × 2W) with either melflufen (*n* = 3) or with saline (*n* = 3). Intraperitoneal fluid samples were drawn at 5, 15, and 30 min each time after IP injection, while blood samples were only drawn once after 30 min. using cardiac puncture followed by euthanasia. After the IP addition of melflufen (73 μg/mL), the concentrations of melflufen, de-ethyl melflufen and melphalan in plasma and IP fluid were analyzed using HPLC-MS/MS at OnTarget Cemistry AB, Uppsala, Sweden. Quantification was performed using deuterium labeled internal standards (IS), D8-melflufen and D8-melphalan.

### Statistical analysis

Statistical analysis was performed with Graphpad Prism™ 6 (Graphpad software, Inc.; La Jolla, Ca, USA). FMCA and MTT IC_50_-values were calculated using non-linear regression analysis (dose-respons inhibition). Single comparisons of appropriate groups were done with Student's *t-test*, multiple comparisons with one-way ANOVA and Turkey's multiple comparison post -test. Data from the *in vivo* IP and SP models were analyzed using non-parametric tests (Kruskall-Wallis and Dunn's multiple comparisons test) and the Mann-Whitney test. Survival analysis was estimated using the Kaplan-Meier method, and survival differences between goups were evaluated using the log-rank test for trend. A *p-value* < 0.05 was considered to indicate statistical significance.
